# Supplementary figures and images for: Development and use of integrated wetland condition index for lacustrine fringe wetlands of Lake Tana, Northwest Ethiopia
Source: PLoS One. 2025 Jun 16;20(6):e0314673. doi: 10.1371/journal.pone.0314673 (PMC12169567; doi:10.1371/journal.pone.0314673)

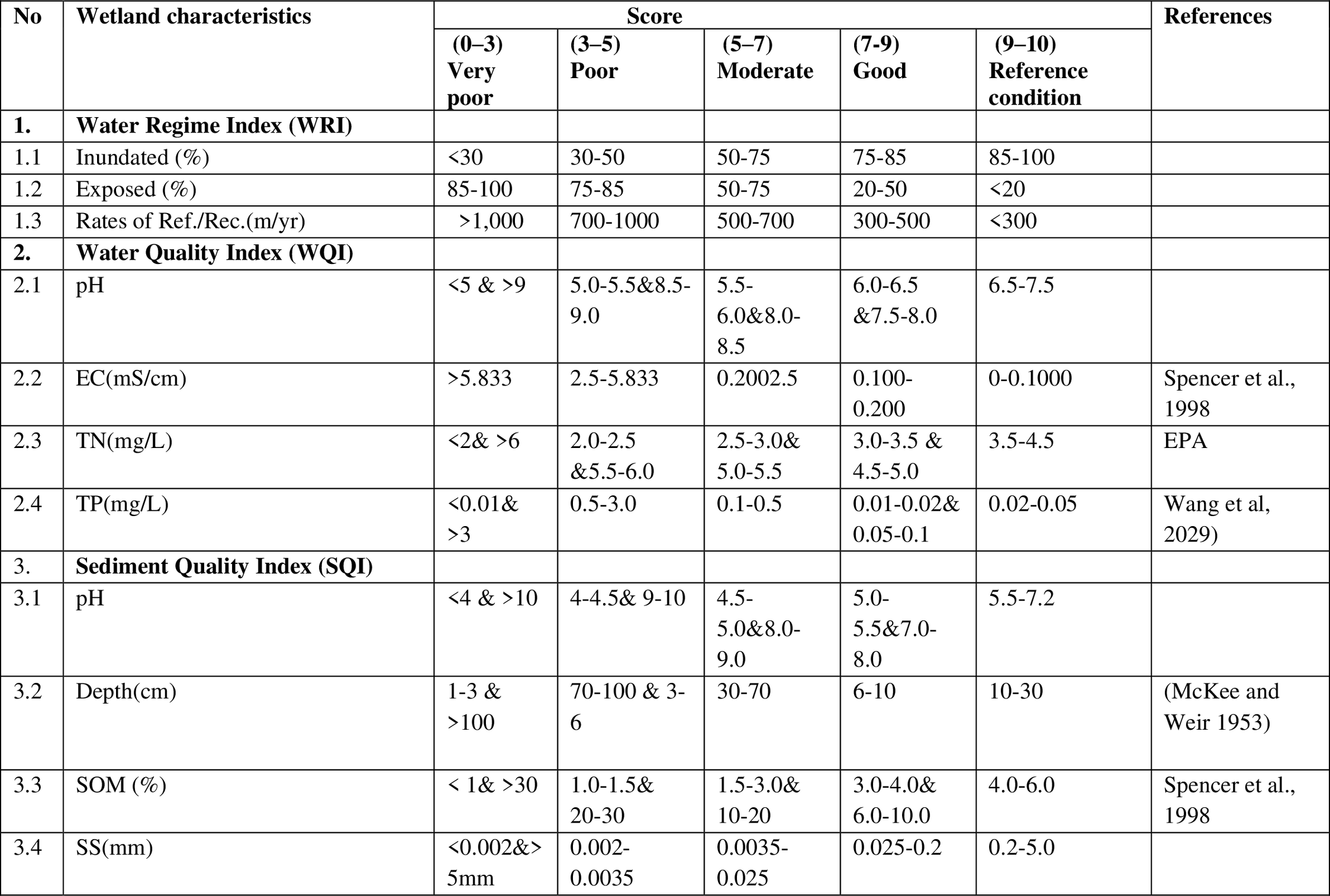

Supplement: S1 Table — (TIF) [file pone.0314673.s001.tif]

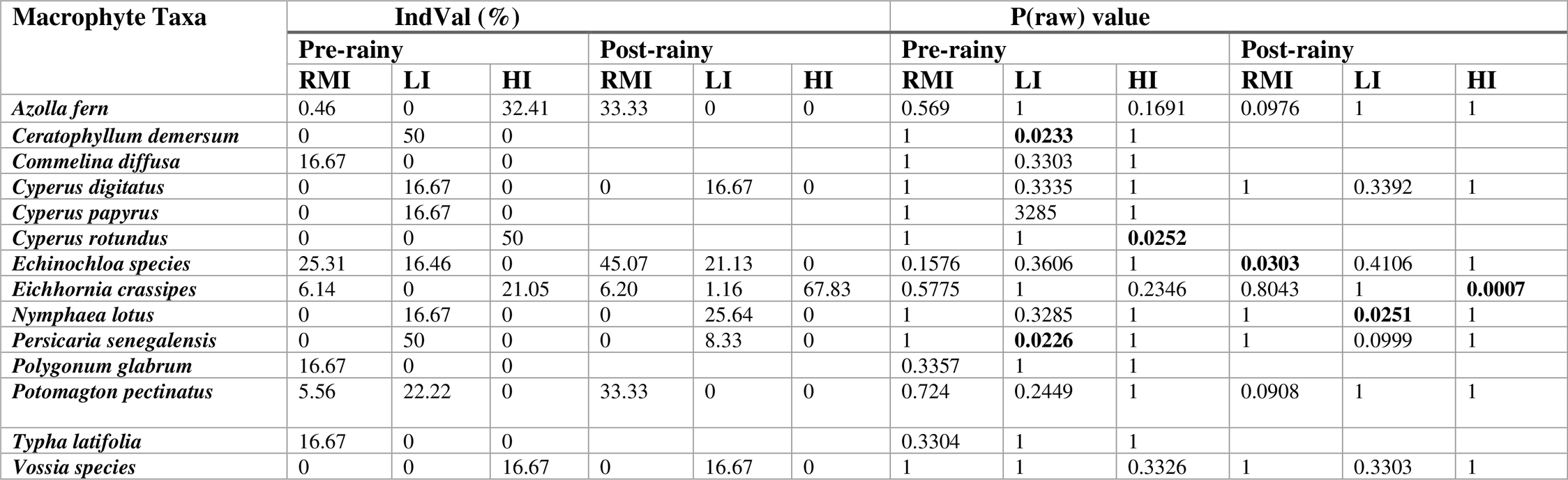

Supplement: S2 Table — Bold values indicate significant P values at p < 0.10. (TIF) [file pone.0314673.s002.tif]

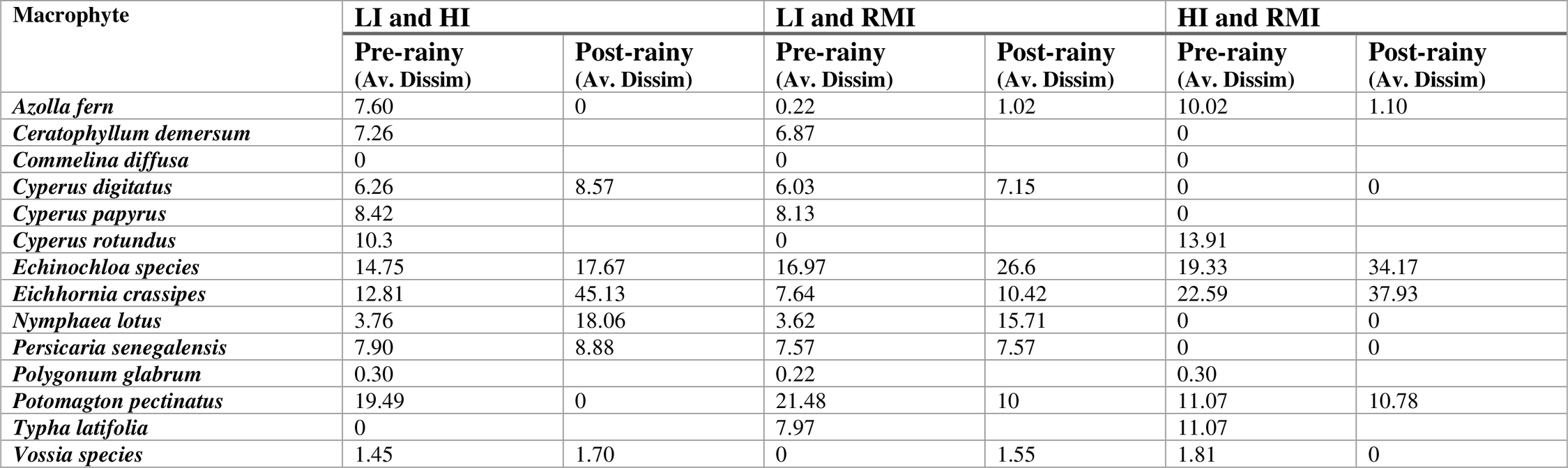

Supplement: S3 Table — Shaded areas indicated macrophyte taxa that did not identified in the given season. (TIF) [file pone.0314673.s003.tif]

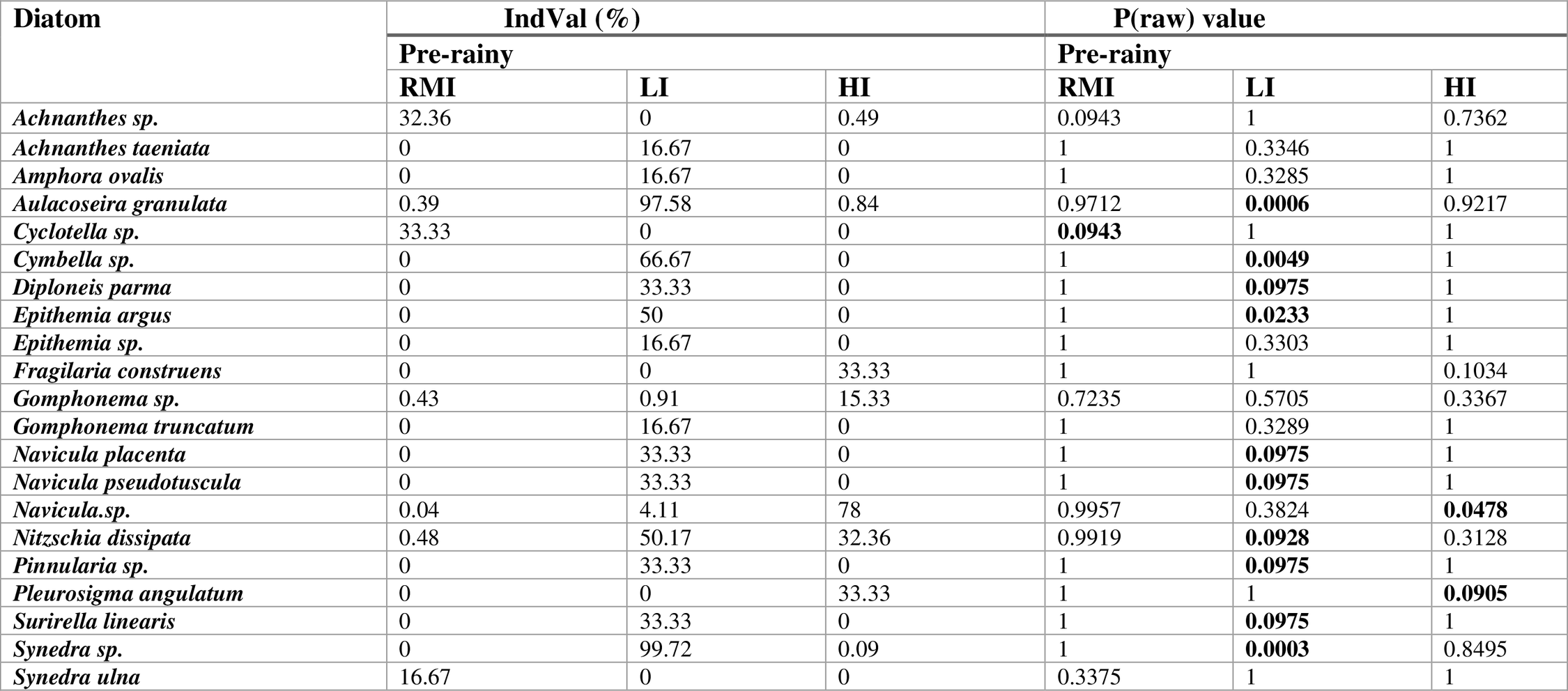

Supplement: S4 Table — Bold values indicate significant at p < 0.10. (TIF) [file pone.0314673.s004.tif]

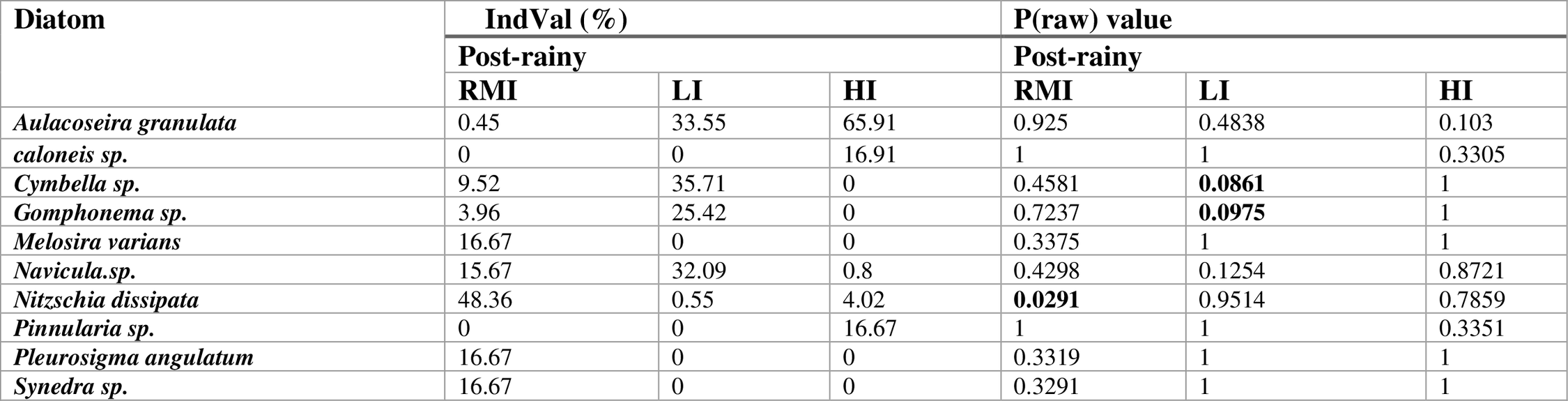

Supplement: S5 Table — Indicator species analysis (ISA) results of diatom species with their IndVal (%) and p value during post-rainy season. (TIF) [file pone.0314673.s005.tif]

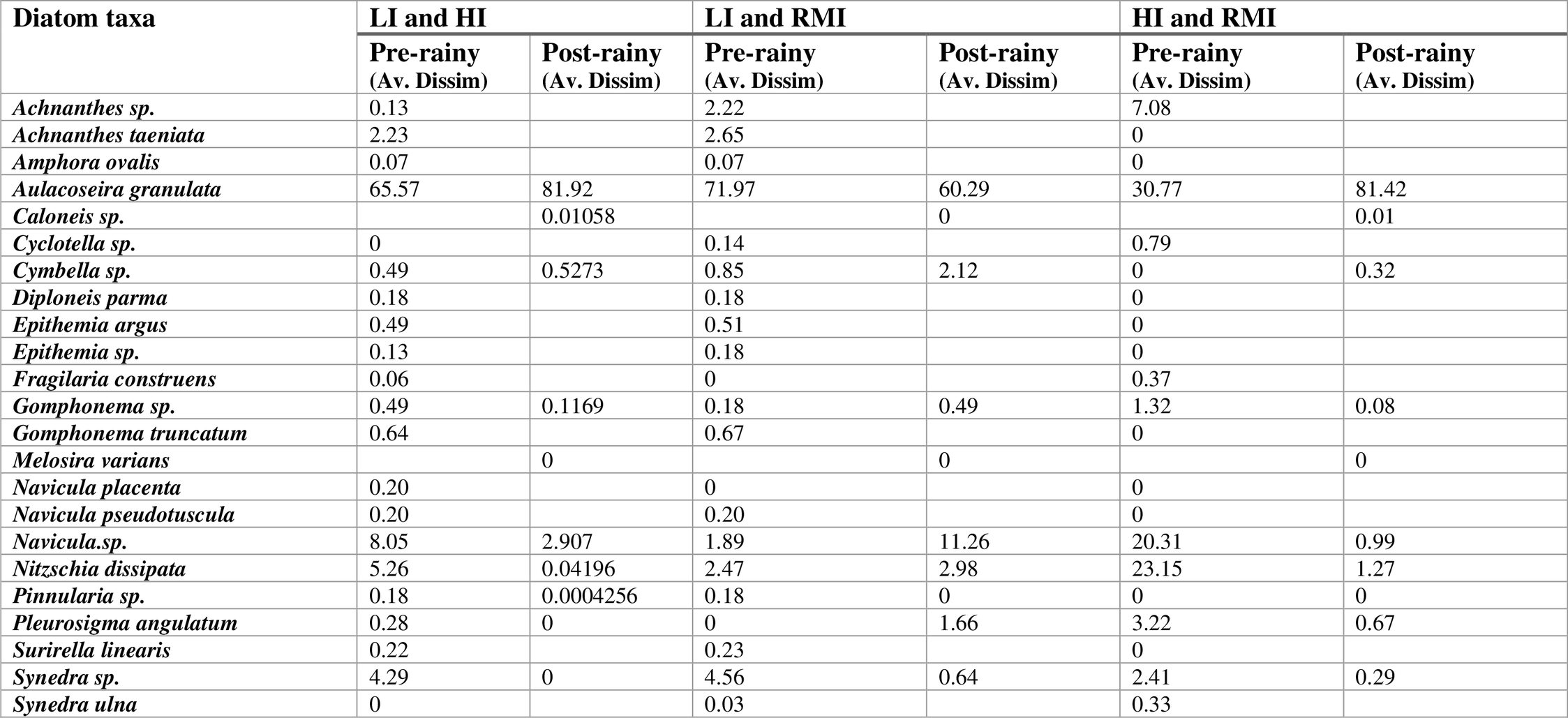

Supplement: S6 Table — Open areas indicated diatom species that did not identified in the given season. (TIF) [file pone.0314673.s006.tif]

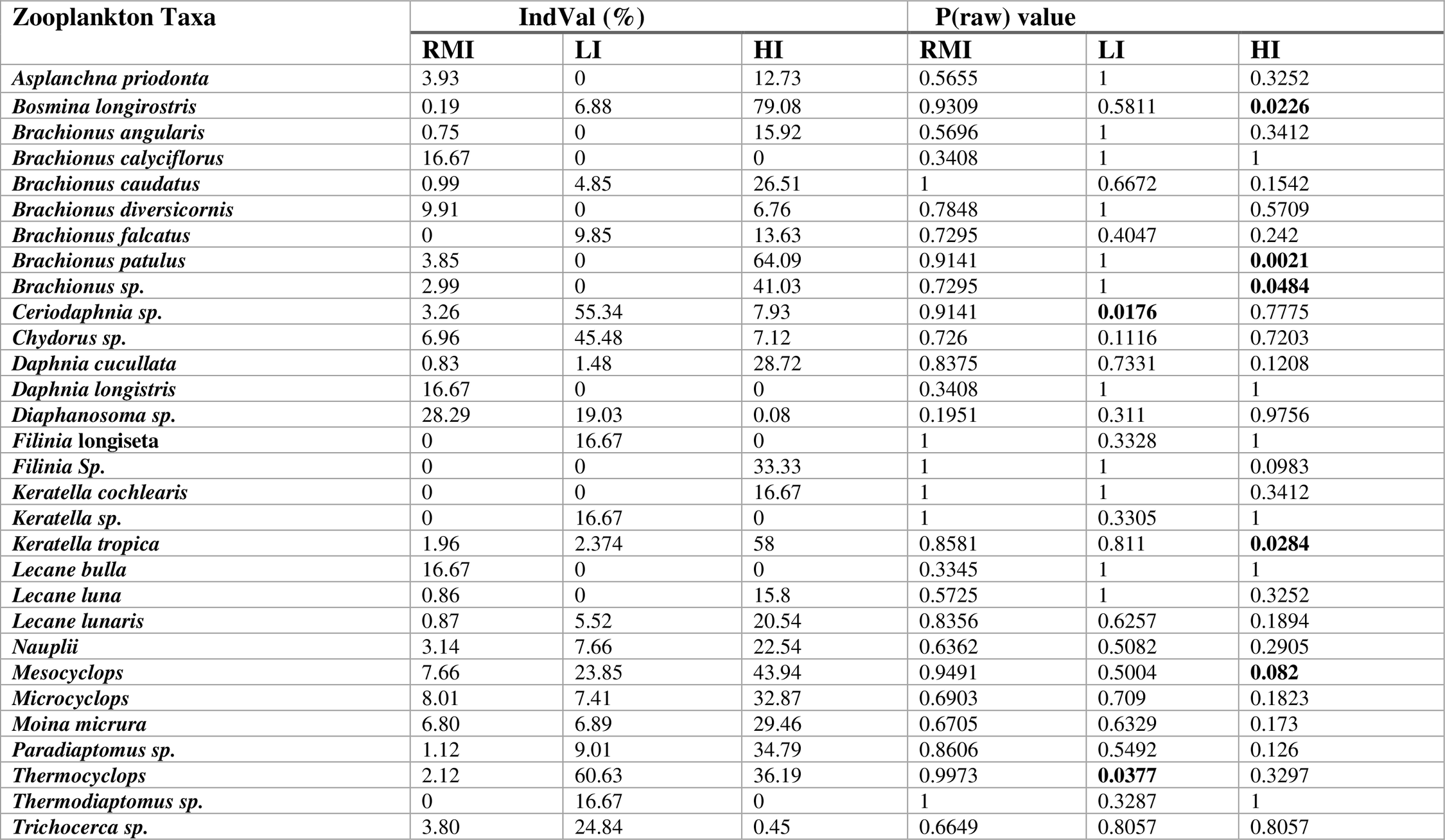

Supplement: S7 Table — Bold values indicate significant P values at p < 0.10. (TIF) [file pone.0314673.s007.tif]

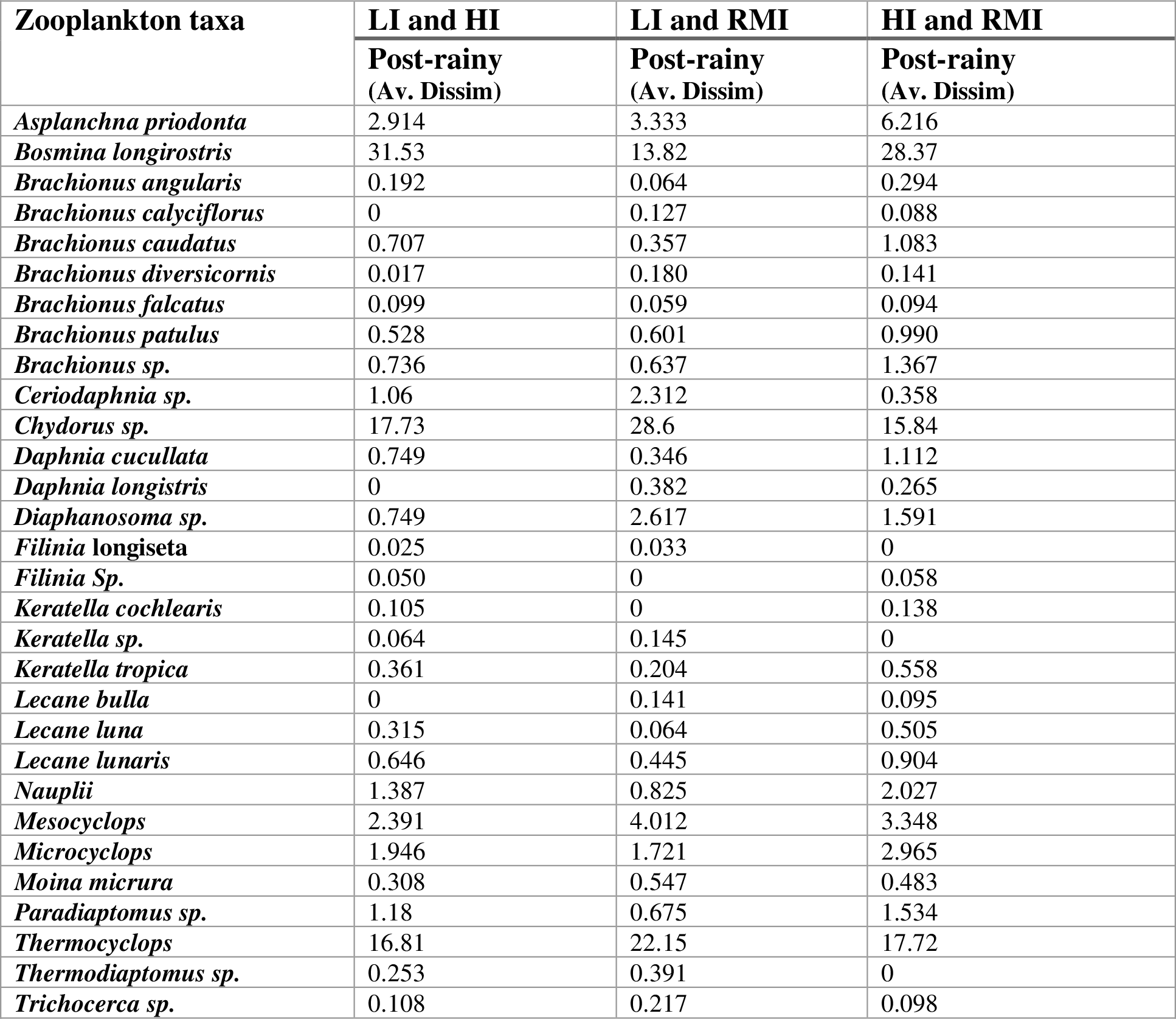

Supplement: S8 Table — (TIF) [file pone.0314673.s008.tif]

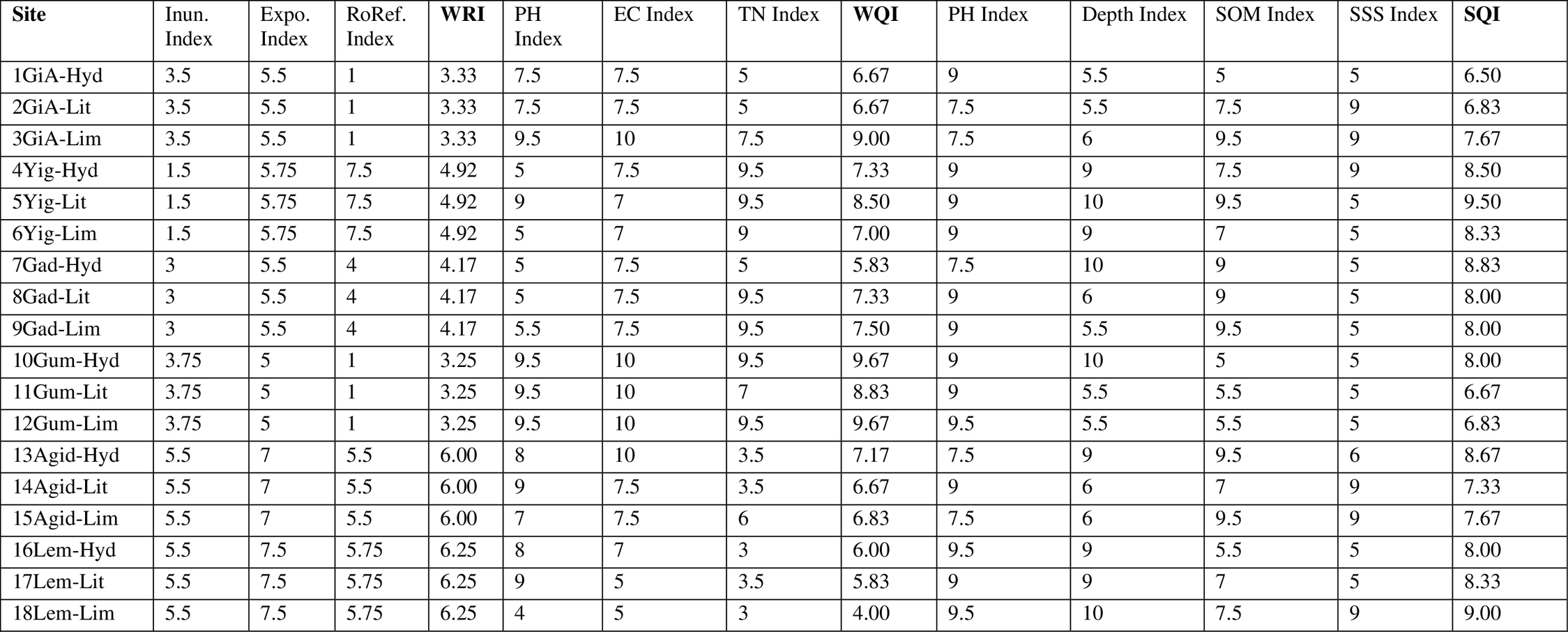

Supplement: S9 Table — (TIF) [file pone.0314673.s009.tif]

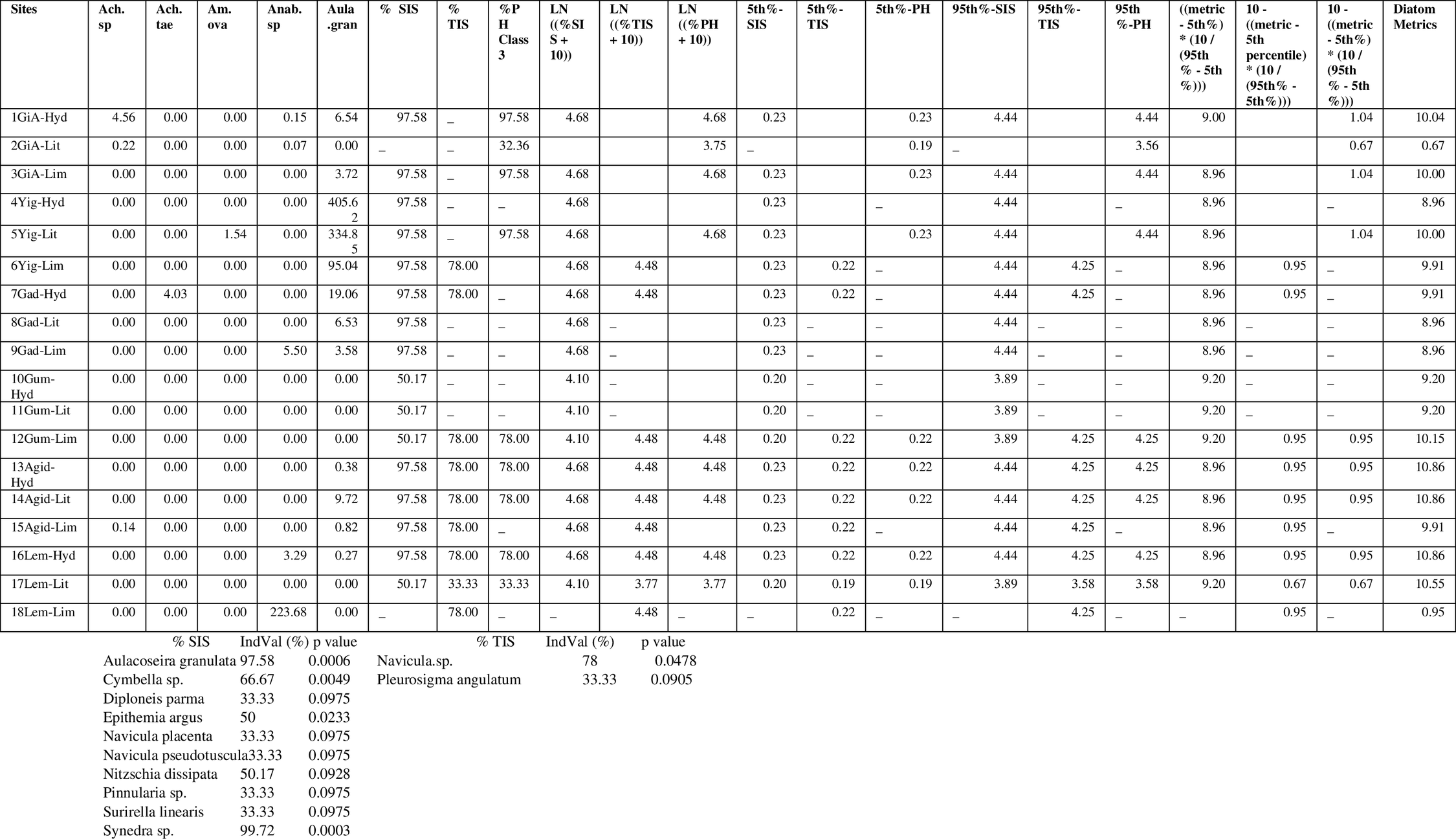

Supplement: S10 Table — (TIF) [file pone.0314673.s010.tif]

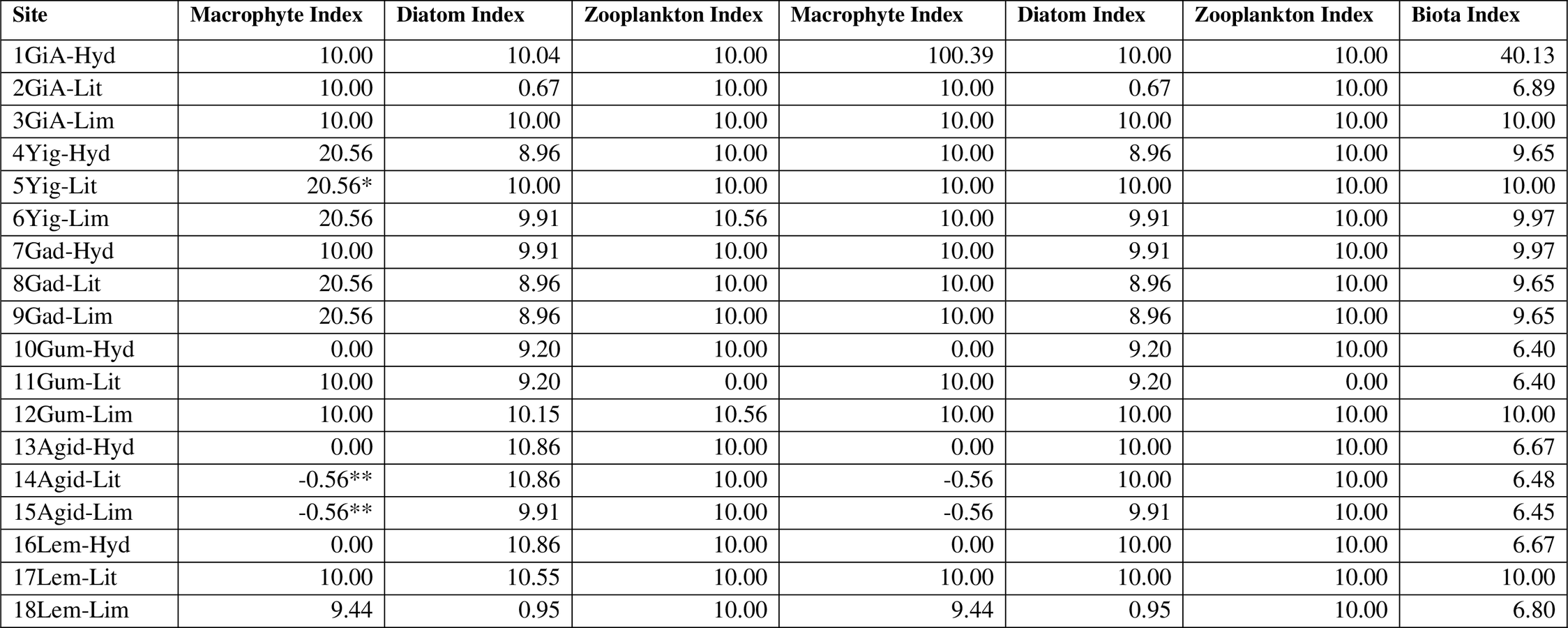

Supplement: S11 Table — Where **values less than 0 are interpreted as 0, and * values more than 10 as 10. (TIF) [file pone.0314673.s011.tif]

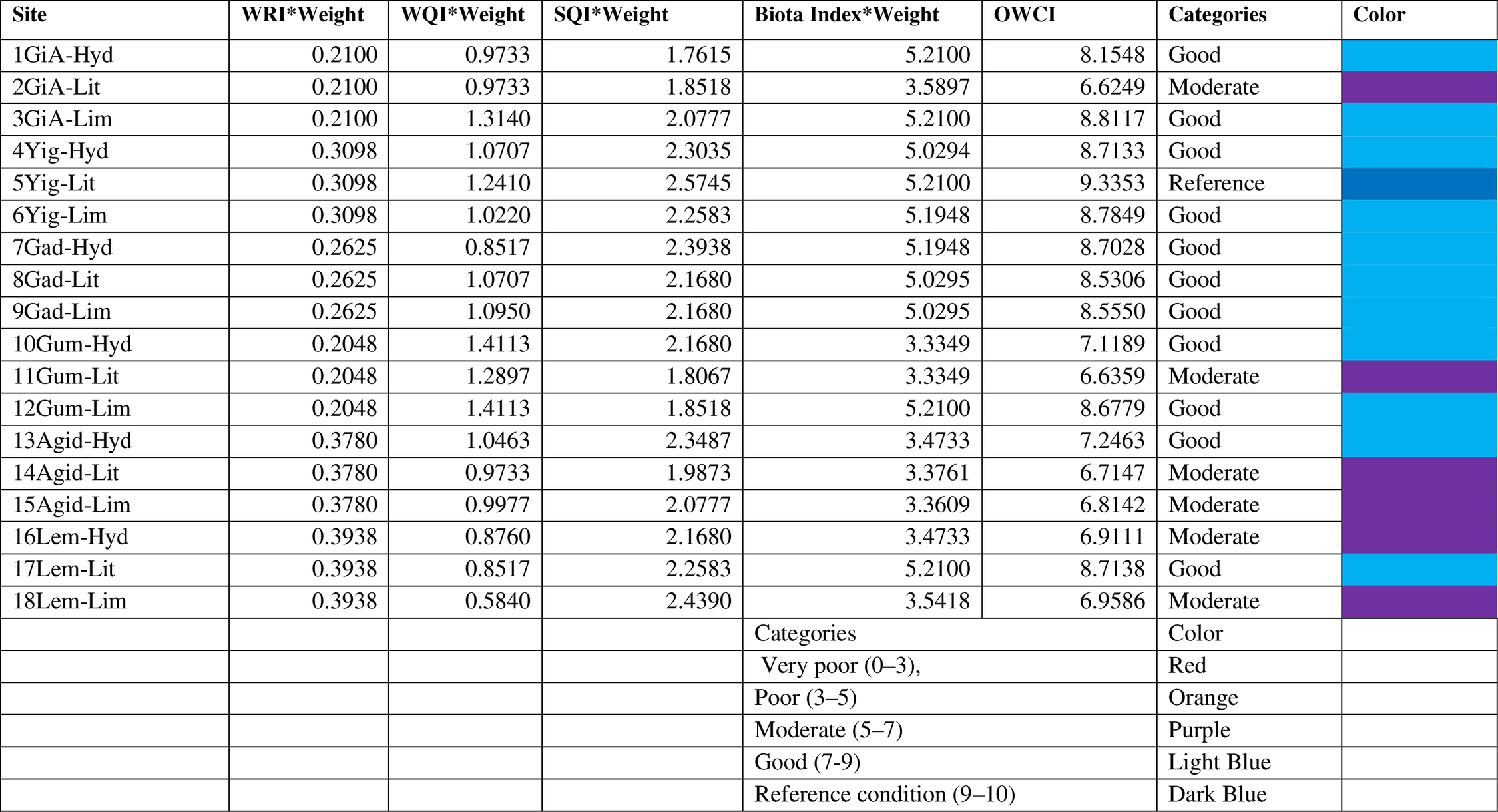

Supplement: S12 Table — (TIF) [file pone.0314673.s012.tif]

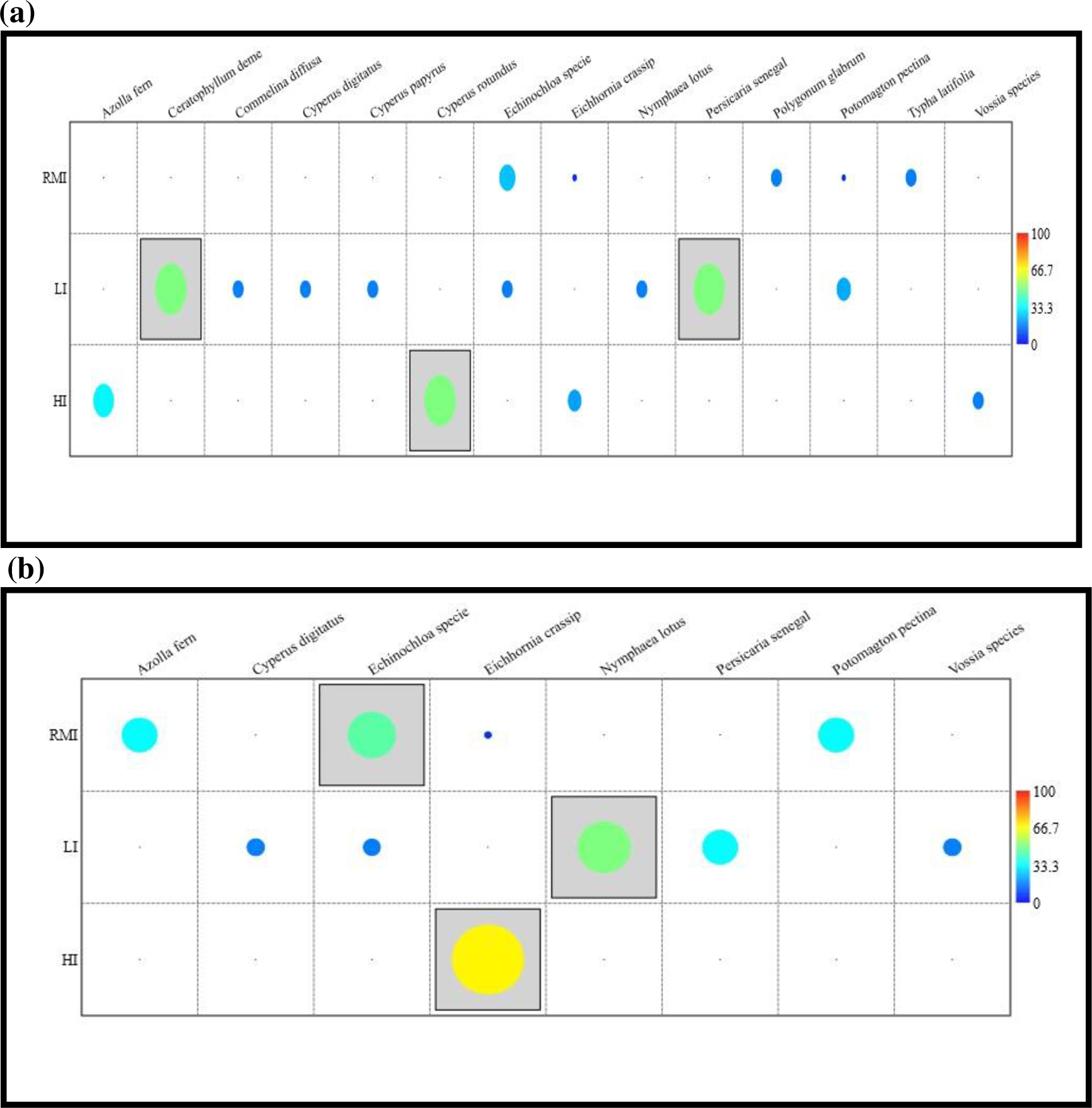

Supplement: S1 Figure — (TIF) [file pone.0314673.s013.tif]

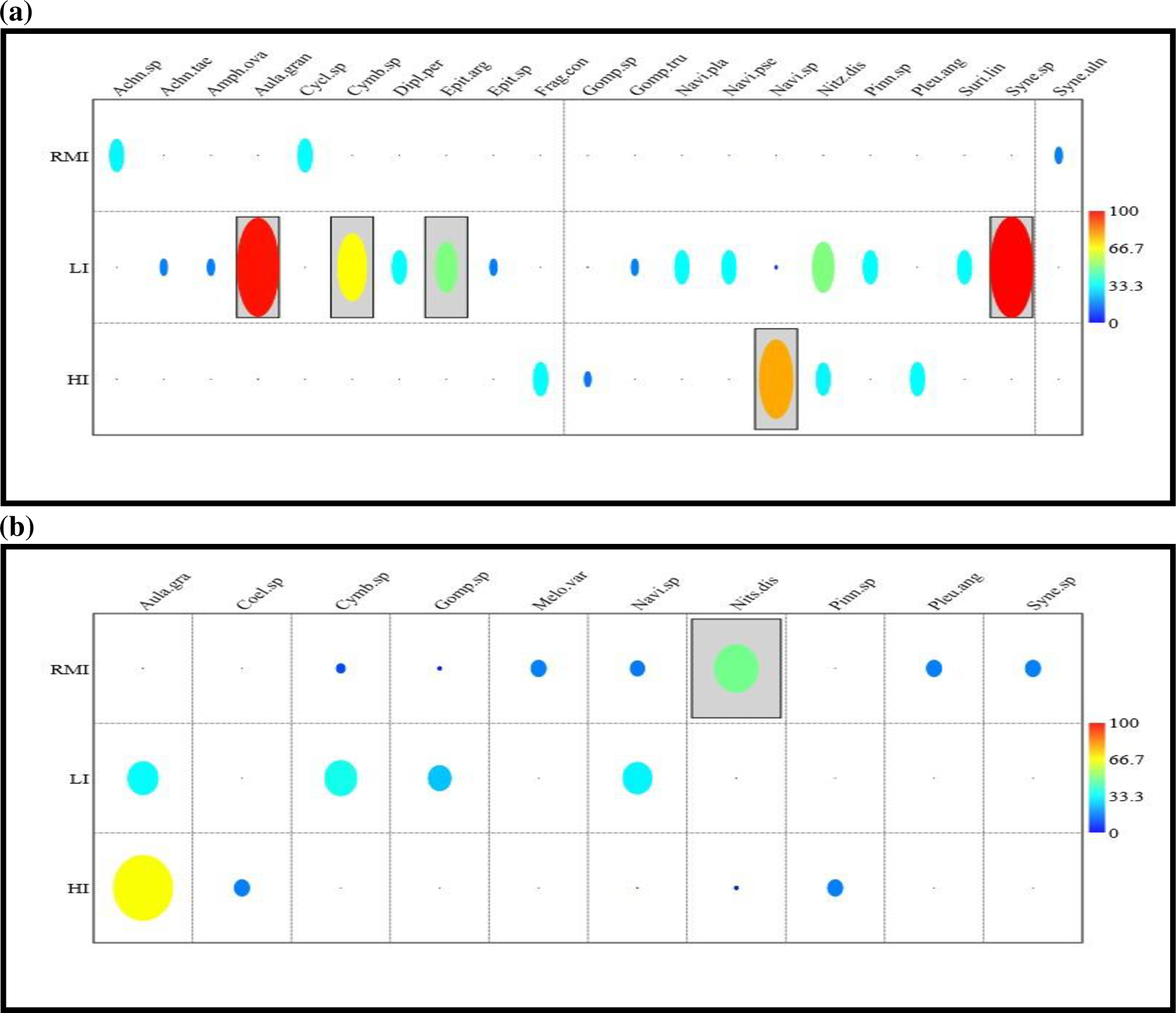

Supplement: S2 Figure — (TIF) [file pone.0314673.s014.tif]

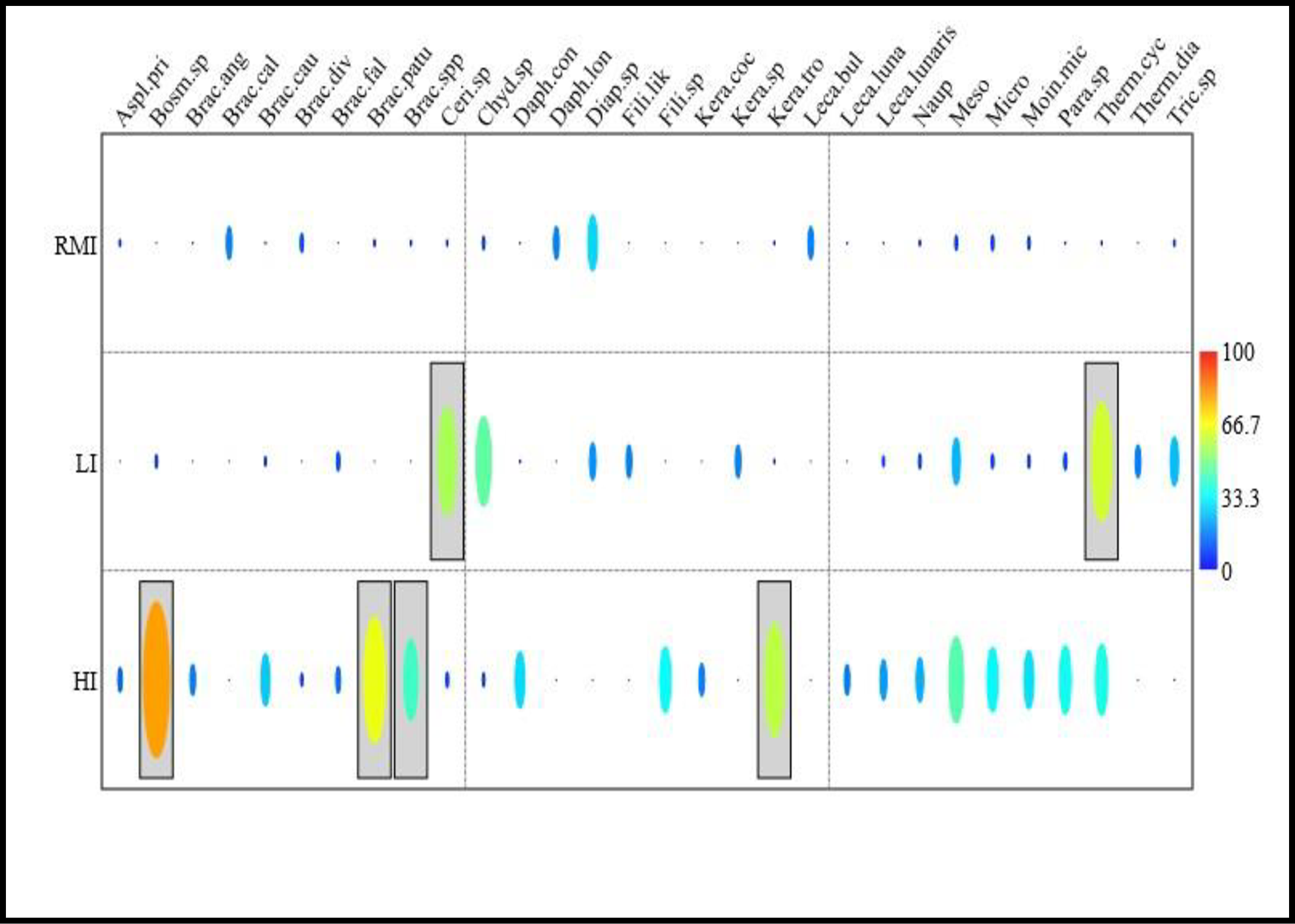

Supplement: S3 Figure — (TIF) [file pone.0314673.s015.tif]
