# Supplementary material for: Development and use of integrated wetland condition index for lacustrine fringe wetlands of Lake Tana, Northwest Ethiopia
Source: PLoS One. 2025 Jun 16;20(6):e0314673. doi: 10.1371/journal.pone.0314673 (PMC12169567; doi:10.1371/journal.pone.0314673)
Supplement: Attachment — Submitted filename: Response to Reviewers.docx [file pone.0314673.s017.docx]

**Response to Reviewers**

| **No.** | **Reviewers-Comments** | **Response to Reviewers** |
| --- | --- | --- |
|  | **Acknowledgements** | The authors wish to thank the anonymous reviewers for their valuable comments and recommendations on the original version of this study. |
|  | **Reviewer-1 Comments** | **Response to Reviewer-1** |
| **1.** | **General Comments:** |  |
|  | Typo mistakes, grammatical and syntax corrections are required in certain places | Thank you for your comments. Typo mistakes, grammatical and syntax errors were corrected. |
| **2** | **Abstract** |  |
|  | The abstract is satisfactory. | Ok! |
| **3.** | **Introduction** |  |
|  | The introduction provides sufficient details and context. | Ok! |
| **4.** | **Materials and Methods:** |  |
| **4.1** | The rationale for choosing specific post-hoc tests, such as Fisher’s LSD, should be explained in more detail. Additionally, it would be beneficial to briefly discuss alternative methods, like Dunn’s test, to help readers understand the reasoning behind the choice. | Fisher’s LSD pairwise comparison method was chosen in this study for the following reasons: (a) Fisher's LSD is essentially a series of individual t tests, in contrast to the Bonferroni, Tukey, Dennett, and Holm methods; instead of computing the pooled SD from just the two groups under comparison, this makes it more difficult to achieve significance; instead, it computes the pooled SD from all the groups, which increases power; (b) Fisher's LSD method for multiple comparisons is used in ANOVA to create confidence intervals for all pairwise differences between factor level means, whereas Dunn's test is the appropriate nonparametric pairwise multiple-comparison procedure when a Kruskal–Wallis test is rejected; and (c) Fisher’s LSD test implemented in the R software using the "agricolae" package, whereas Dunn's test is implemented for Stata in the dunntest command. |
| 4.2 | More clarity is needed regarding the specific statistical functions used in each statistical package. This will improve the reproducibility of the analysis by other researchers. It would be helpful to explicitly mention which software and versions were used, and provide clear references to the specific functions applied for each part of the analysis. | Software’s of R version 4.1.3 (2022-03-10) and PAST version 4.14 were used. Fisher’s LSD test was performed using the R package "agricolae”. For complete functionality of agricolae package, other packages such as MASS, nlme, klaR, Cluster, and algDesign were installed and loaded. Indicator species tests (IndVal% and p (raw)), ‘Analysis of Similarities’ (ANOSIM), and ‘Similarity Percentages’ (SIMPER) performed in PAST 4.14. |
| 4.3 | While the overall framework for the scoring system is clear, more detail is required on the rationale behind the system, including the criteria for weighting different components. | For instance, 52.10% of the weight was assigned to Wetland Biota Index followed by 27.10% and 14.60 % to Water Quality Index (WQI), and Sediment Quality Index (SQI) respectively whereas, Water Regime Index (WRI) were assigned the least weights of 6.30%.Generally, our study result indicated that variations in Environmental variables (hydrology, water and soil) were weaker than biotic variables. Because except very low water level phase, lacustrine fringe wetlands hydrologically connected with Riverine Wetlands, floodplains & the Lake Tana itself. This hydrological process facilitated the exchange of water, sediments, nutrients & organisms and leads to low spatial environmental heterogeneity. |
| 4.4 | How was the accuracy of the Integrated Lake Wetland Condition Index (ILWCI) validated? Providing details on the validation process will enhance the credibility of the index. Further explanation of how the indices were validated would improve the transparency of the method. | Thank you for you critical comments of validation of IWCI. As per your comments validation of IWCI included both in the methodology, result/discussion and conclusion/suggestion sections.  **Materials and methods**  Overall wetland condition index calculation and validation. Correct classification percentage (CCP) [47] used to measure precision. A CCP higher than or equal to 70% is considered as reliable in ecological applications [48].  **Results**  Among the 18 samples only 10 were correctly classified by the integrated wetland index (CCP = 55.55%). The CCP value of this study is below 70%. This indicates that IWCI of high water level period is not considered as reliable in ecological applications [48]. IWCI does not discriminate reference from impaired sites. However as compare to IWCI of low water level period, IWCI of high water level period is better to discriminated the impacted wetland categories from the reference wetland categories. The selection of sites, as reference based on a prior knowledge of human disturbance impact pressures could not identify the exact reference sites. Thus, a post classification based on human disturbance score of [31] might could select the exact reference sites. Therefore, for future research works on the development of wetland condition index, reference and impacted sites needs to be selected based on a post classification approach than a prior knowledge of human disturbance impact pressures.  **Conclusion**  The precise reference sites could not be identified since the selection of sites was based on a prior understanding of the effect pressures caused by human disturbances. Therefore, reference and impacted sites needs to be chosen using a post-classification approach rather than a prior knowledge of the impact pressures caused by human disturbances for future research projects on the development of the wetland state index. |
| 4.5 | To improve transparency and interpretation, it would be helpful to include a data dictionary or a table summarizing the raw values and scoring systems for each parameter. This would allow readers to better understand the scoring process and the data being used. | Thank you for you critical comments. Data dictionary or a table summarizing the raw values and scoring systems for each four wetland components and overall WICI included under supplementary materials:  Supplementary Table 9 Summarized the raw values of environmental and biota data, scoring systems for each parameter and overall integrated wetland index.  Supplementary Table 9.1 Summarized scoring values of water regime, water quality and sediment quality sub-indices  Supplementary Table 9.2 Sample diatom species normalize score values between 0 and 10 using [46] equation  Supplementary Table 9.3 Summary of Macrophytes, Diatom, Zooplankton and Overall biota index values  Supplementary Table 9.4 Multiplying each sub-index score by its respective final weight and summing the scores of overall wetland condition index. |
| **5.** | **Results and Discussion:** |  |
| 5.1 | Figure 1: This figure needs improvement. Enhancing the clarity, labels, and possibly adding more detail will make it more informative and easier to interpret. | Academic Editor of PLOS ONE also suggested that while revising your submission; please upload your figure files to the Preflight Analysis and Conversion Engine (PACE) digital diagnostic tool, https://pacev2.apexcovantage.com/. PACE helps ensure that figures meet PLOS requirements. Now we uploaded our figure files using Preflight Analysis and Conversion Engine (PACE) digital diagnostic tool and enhanced the resolution of the figures. |
| 5.2 | Figure 2: The resolution of this figure should be improved to ensure that all details are clear and legible. | The same with 5.1 response for comments |
| 5.3 | The discussion section would benefit from an acknowledgment of the limitations of the methods used in the study as well as the potential limitations of the proposed Index. | Please see the last paragraph of the discussion section.  Although this IWCI developed and tested in this study could be a tool to inform decision-makers on lacustrine wetland conservation and restoration priorities throughout the Lake Tana Ecosystem, there are some limitations of the methods used in the study as well as the proposed index and future prospects of the study that needed to be strengthened. Factors that limit the development and test of IWCI for lacustrine fringe wetlands in Lake Tana were (a) limitations in spatial scale (only six selected wetlands were not sufficiently representative of the entire lacustrine wetland in Lake Tana). (b) limitation in temporal scale (the study was conducted only during two seasons: the pre-rainy (drawdown phase) of May 2021 and the post-rainy (post-flooding phase, or PFP) of October 2021, i.e., the rest of the dry season of water level declining (recession phase) and the rainy season of water level rising (refill phase) were not covered). (c) IWCI does not sufficiently discriminate reference from impaired sites (CCP value of below 70%). Low CCP value could be associated with the limitation of the prior knowledge classification approach. The precise reference sites could not be identified since the selection of sites was based on a prior understanding of the effect pressures caused by human disturbances. This could influence the overall index value. Therefore, reference and impacted sites need to be chosen using a post-classification approach rather than prior knowledge of the impact pressures caused by human disturbances for future research projects on the development of the wetland state index. (d) Indicators at the landscape level, wetland biota of fish, and wetland birds as indicator species were not included in this research work. Thus, further research needs to be done by including landscape, fish, birds, and long-term water level variability indicators in the future. |
| **6.** | **Conclusions:** |  |
|  | The conclusions are satisfactory. | **Ok!** |
| **7.** | **Supplementary Materials:** |  |
|  | All figures in the Supplementary needs enhancement, the figures should be readable and in clear resolution | All figures in the Supplementary enhanced. |
|  | **Reviewer-2 Comments** | **Response to Reviewer-2** |
| **1.** | **General Comment:** |  |
|  | In the manuscript, an integrated wetland condition index (IWCI) system was tried to develop for lacustrine for a wetland in Ethiopia based on the four conditions of the existing wetlands hydrology, chemistry, water quality and biota using some sort of simulation modelling. The research is appealing, but the quality of information, applied methodologies and findings have major flaws, which need to be validated before the final decision. Some of the specific comments are as follows: | Thank you for you critical comments of validation of IWCI. As per your comments validation of IWCI included both in the methodology, result/discussion and conclusion/suggestion sections.  **Materials and methods**  Overall wetland condition index calculation and validation. Correct classification percentage (CCP) [47] used to measure precision. A CCP higher than or equal to 70% is considered as reliable in ecological applications [48].  **Results**  Among the 18 samples only 10 were correctly classified by the integrated wetland index (CCP = 55.55%). The CCP value of this study is below 70%. This indicates that IWCI of high water level period is not considered as reliable in ecological applications [48]. IWCI does not discriminate reference from impaired sites. However as compare to IWCI of low water level period, IWCI of high water level period is better to discriminated the impacted wetland categories from the reference wetland categories. The selection of sites, as reference based on a prior knowledge of human disturbance impact pressures could not identify the exact reference sites. Thus, a post classification based on human disturbance score of [31] might could select the exact reference sites. Therefore, for future research works on the development of wetland condition index, reference and impacted sites needs to be selected based on a post classification approach than a prior knowledge of human disturbance impact pressures.  **Conclusion**  The precise reference sites could not be identified since the selection of sites was based on a prior understanding of the effect pressures caused by human disturbances. Therefore, reference and impacted sites needs to be chosen using a post-classification approach rather than a prior knowledge of the impact pressures caused by human disturbances for future research projects on the development of the wetland state index. |
| **2.** | **Abstract** |  |
|  | Please rewrite the abstract with this flow: issue/purpose, methodology applied, major findings, concluding remarks with a main recommendation. In its present form, it is very segregated and could not get the outcomes. | We rewrote the manuscript as per your comments by taking into account: issue/purpose, methodology applied, major findings, concluding remarks with a main recommendation (see revised Abstract with trach changes. |
| **3.** | **Introduction** |  |
| **3.1** | Literature presented in the manuscript must be cited from the recently published literature. Still there information regarding the decision-making system is missing in the existing literature. Gap analysis needs to be clearly mentioned at the last of the introduction session. | Please see the gap analysis that mentioned at the last of the introduction session (gap analysis at the national level).   (a) Gap analysis at the international level   Few studies have focused on integrated wetland condition indices. In order to manage and conserve wetlands, there is a need for integrated methods of assessment [13]. Recently, efforts have been made to combine and alter a few indices to develop an integrative expression that considers different environmental components. For environmental assessment of wetlands, [15] integrated hydrogeomorphic and Index of Biotic Integrity approaches into a single, more flexible, and broadly based approach. For Victoria, Australia, [16] developed a wetland condition index by integrating catchments, physical form, soils, water chemistry, hydrology, and biota for Victoria, Australia. The Landscape Development Intensity Index (LDI) and the Water Environment Index (WEI) were combined by [17] to create the Wetland Condition Index (WCI) for China's humid regions.   (b) Gap analysis at the national level   In the case of Ethiopia, monitoring of freshwater ecosystem health has so far focused on macroinvertebrates and, to a lesser extent, on diatoms [22] . In Ethiopia, the use of macroinvertebrates has increased in recent times [22–26] . Biomonitoring of freshwater ecosystem health using diatoms and plants as indicator species in East Africa, including Ethiopia, are very limited. [27,28] used diatoms to monitor the ecosystem health of rivers in Ethiopia, while [29] developed a multimetric plant-based index of biotic integrity for assessing the ecological state of forested, urban, and agricultural natural wetlands in Jimma Highlands, Ethiopia. **However, an integrated wetland condition index is not yet developed for monitoring freshwater ecosystem health in Ethiopia.**  Consequently, this research aimed to develop an integrated wetland condition index (IWCI) for lacustrine fringe wetlands (LFWs) in Lake Tana, Northwest Ethiopia based on characteristics and components that define wetlands, literature reviews, and the data collection and analysis of this research project. |
| 3.2 | Is this the only study done of this specific wetland? There is no other published literature mentioned in the present manuscript. | Development integrated wetland condition index for lacustrine fringe wetlands of Lake Tana, Northwest Ethiopia is the only study of this specific wetland. However, there are other published research results on this wetland and included in this study.  These published research results cited to discuss or compare the present wetland water chemistry conditions and wetland biota assemblages.  **Wetland water chemistry conditions in comparison with past data**  In comparison with past data of water chemistry, the observed pH values (pH = 6.58–8.64) during low water level and (pH =7.40–9.98) during high water level almost fell within the pH range (pH =7.6–8.9) reported by [50]. The range value of the water temperature (18.20–32.10°C) has a wider range in this study as compared to the range value (24.15–26.34°C) reported by [50].The mean EC values of 133.6 µS/cm and 138 µS/cm during low and high water levels, respectively, of this study fell within the EC range (152.48-164.18 µS/cm) and (111-305 µS/cm) reported by [50, 51], respectively. The values of total phosphorus in water (TP = 0.23–2.39 mg/l) during low water level and (TP = 2.02–3.14 mg/l) during high water level of this study are higher than those reported by [50] total phosphorus in water (TP = 0.22–1.00 mg/l) and [52] total phosphorus in water (TP = 0.44–1.73 mg/l). The absence of well-established buffer zones and nutrient enrichment of the lake's littoral zone from human sources in the catchment area are likely to be responsible for the rising trend in total nitrogen and total phosphorus in the water. The rising trend in nutrient levels is closely linked to runoff from agriculture and sewage from homes and businesses that is dumped into lake[53,50].  **Wetland biota assemblages in comparison with past data**  A total of 14 macrophyte species were identified in this study, twice as small as the macrophyte species (30) identified by [50]. Almost all macrophyte species reported both during low and high water level phases were listed in the study of [50,54]. The macrophyte species diversity of shore area wetlands in Lake Tana is low compared with the previous works of [50].This could be related to the expansion of free-floating species invasive species of Eichhornia crassipes (water hyacinth).The total number of zooplankton taxa in our study is smaller than a total of 57 zooplankton taxa representing Cladocerans (24 taxa), Copepods (9 species), and Rotifers (24 taxa) by [55].However, zooplankton taxa richness in our wetlands (31 zooplankton taxa) was higher than the review work of [56] who reviewed that several zooplankton studies in Lake Tana found a total of 26 zooplankton species, including 3 copepods, 11 cladocerans, and 12 rotifers. The total number of zooplankton taxa reported in the current study was also higher; a total of 13 species, four copepods, and nine cladocerans were identified by [57]. |
| 4. | **Materials and Methods:** |  |
| 4.1 | Why only these few parameters are considered to be investigated? There are other important parameters too? | Macroinverteberate data were collected. However, the reliability of the data was not reliable. So we decided to exclude from this study. Indicators at the landscape level, wetland biota of fish, and wetland birds as indicator species were not included in this research work. Thus, further research needs to be done by including landscape, fish, birds, and long-term water level variability indicators in the future. |
| 4.2 | The sampling procedure needs to be given in details. Why could be the sources of these nutrients? | Sampling procedures presented in details. Additional procedures included after your comments and suggestions. |
| 4.3 | How the information or data is reliable and reflect for the whole year? The sampling is performed once in a year. | The sampling is performed twice in a year. Limitation in temporal scale (the study was conducted only during two seasons: the pre-rainy (drawdown phase) of May 2021 and the post-rainy (post-flooding phase, or PFP) of October 2021, i.e., the rest of the dry season of water level declining (recession phase) and the rainy season of water level rising (refill phase) were not covered). |
| 4.4 | Continue monitoring can provide the overall conditions of the wetland health and this is one of the missing point in the present study. | Limitation in temporal scale (the study was conducted only during two seasons: the pre-rainy (drawdown phase) of May 2021 and the post-rainy (post-flooding phase, or PFP) of October 2021, i.e., the rest of the dry season of water level declining (recession phase) and the rainy season of water level rising (refill phase) were not covered). |
